# Hypomorphic variants of SEL1L-HRD1 ER-associated degradation are associated with neurodevelopmental disorders

**DOI:** 10.1172/JCI170054

**Published:** 2024-01-16

**Authors:** Huilun H. Wang, Liangguang L. Lin, Zexin J. Li, Xiaoqiong Wei, Omar Askander, Gerarda Cappuccio, Mais O. Hashem, Laurence Hubert, Arnold Munnich, Mashael Alqahtani, Qi Pang, Margit Burmeister, You Lu, Karine Poirier, Claude Besmond, Shengyi Sun, Nicola Brunetti-Pierri, Fowzan S. Alkuraya, Ling Qi

**Affiliations:** 1 Department of Molecular Physiology and Biological Physics, University of Virginia School of Medicine, University of Virginia, Charlottesville, Virginia, USA.; 2 Department of Molecular & Integrative Physiology and; 3 Department of Biological Chemistry, University of Michigan Medical School, Ann Arbor, Michigan, USA.; 4 Hopital Cheik Zaïd, Hopital Universitaire International RABAT, Morocco.; 5 Telethon Institute of Genetics and Medicine, Pozzuoli, Italy.; 6 Department of Translational Medicine, University of Naples Federico II, Naples, Italy.; 7 Department of Translational Genomics, Center for Genomic Medicine, King Faisal Specialist Hospital and Research Center, Riyadh, Saudi Arabia.; 8 Imagine Institute, INSERM UMR1163, Paris, France.; 9 Université Paris Cité, Paris, France.; 10 Department of Neurosurgery, Shandong Provincial Hospital, Cheeloo College of Medicine, Shandong University, Jinan, Shandong, China.; 11 Michigan Neuroscience Institute and Departments of Computational Medicine & Bioinformatics, Psychiatry, and Human Genetics, University of Michigan Medical School, Ann Arbor, Michigan, USA.; 12 Department of Pharmacology, University of Virginia, Charlottesville, Virginia, USA.; 13 Scuola Superiore Meridionale (SSM, School of Advanced Studies), Genomics and Experimental Medicine Program, University of Naples Federico II, Naples, Italy.; 14 Department of Pediatrics, Prince Sultan Military Medical City, Riyadh, Saudi Arabia.

**Keywords:** Cell Biology, Genetic variation, Neurological disorders, Protein misfolding

## Abstract

Recent studies using cell type–specific knockout mouse models have improved our understanding of the pathophysiological relevance of suppressor of lin-12-like–HMG-CoA reductase degradation 1 (SEL1L-HRD1) endoplasmic reticulum–associated (ER-associated) degradation (ERAD); however, its importance in humans remains unclear, as no disease variant has been identified. Here, we report the identification of 3 biallelic missense variants of *SEL1L* and *HRD1* (or *SYVN1*) in 6 children from 3 independent families presenting with developmental delay, intellectual disability, microcephaly, facial dysmorphisms, hypotonia, and/or ataxia. These *SEL1L* (p.Gly585Asp, p.Met528Arg) and *HRD1* (p.Pro398Leu) variants were hypomorphic and impaired ERAD function at distinct steps of ERAD, including substrate recruitment (SEL1L p.Gly585Asp), SEL1L-HRD1 complex formation (SEL1L p.Met528Arg), and HRD1 activity (HRD1 p.Pro398Leu). Our study not only provides insights into the structure-function relationship of SEL1L-HRD1 ERAD, but also establishes the importance of SEL1L-HRD1 ERAD in humans.

## Abstract 2

ER-associated degradation-associated neurodevelopmental disorder with onset in infancy is associated with hypomorphic variants of SEL1L and HRD1.

## Graphical abstract

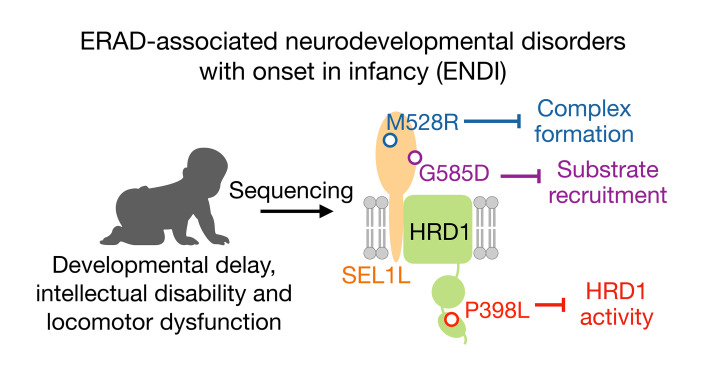

## Introduction

Nascent membrane or secretory proteins are synthesized and folded in the endoplasmic reticulum (ER), which is prone to misfolding. Such misfolding may have pathogenic consequences if not cleared effectively (1-5). The suppressor of lin-12-like–HMG-CoA reductase degradation 1 (SEL1L-HRD1) complex represents one of the most conserved quality-control mechanisms in the cell, known as ER-associated degradation (ERAD) (6–8). In SEL1L-HRD1 ERAD, misfolded proteins are recognized and recruited to the SEL1L-HRD1 protein complex via ER chaperones such as osterosarcoma amplified 9 (OS9) and ER lectin 1 (ERLEC1, also known as XTP3B) (9–14) followed by retrotranslocation and polyubiquitination by the E3 ligase HRD1 (12, 15, 16) and proteasome degradation in the cytosol (17, 18). In this complex, SEL1L is an obligatory cofactor for the E3 ligase HRD1 (19, 20), not only controlling the protein stability of HRD1 (6, 19, 20), but functioning as a scaffold for other ERAD components such as OS9, ERLEC1, and degradation in ER (DERLIN) proteins (10, 19, 21–27). In yeast, SEL1L homolog Hrd3p may regulate Hrd1p autoubiquitination and self-degradation (28, 29).

Global or acute deletion of *Sel1L* or *Hrd1* in germline and adult mice causes embryonic or premature lethality, respectively (20, 30–32), pointing to the requirement of SEL1L-HRD1 ERAD function at both embryonic developmental and adult stages. Subsequent studies using cell type–specific gene KO mouse models have established its vital importance in many physiological processes, including food intake, water balance, thermogenesis, energy homeostasis, gut homeostasis, β cell identity/function, immune cell development/function, and hematopoietic cell quiescence (2, 3, 5, 33–51). Although these advances in mouse models have provided critical insights into the physiological importance of this complex, its relevance in humans remains unknown, as no disease variant has been identified in humans.

Using whole-exome sequencing (WES), here we report the identification of 3 autosomal recessive variants, SEL1L p.Gly585Asp, p.Met528Arg, and HRD1 p.Pro398Leu, in 6 children from 3 unrelated families with similar neurodevelopmental disorders — termed ERAD-associated neurodevelopmental disorders with onset in infancy (ENDI). These variants are hypomorphic and attenuate ERAD function, likely via distinct mechanisms, including substrate recruitment, SEL1L-HRD1 complex formation, and HRD1 activity. Hence, this study establishes the pathophysiological importance of SEL1L-HRD1 ERAD in humans.

## Results

### Identification of biallelic SEL1L and HRD1 variants in humans.

**Figure 1:**
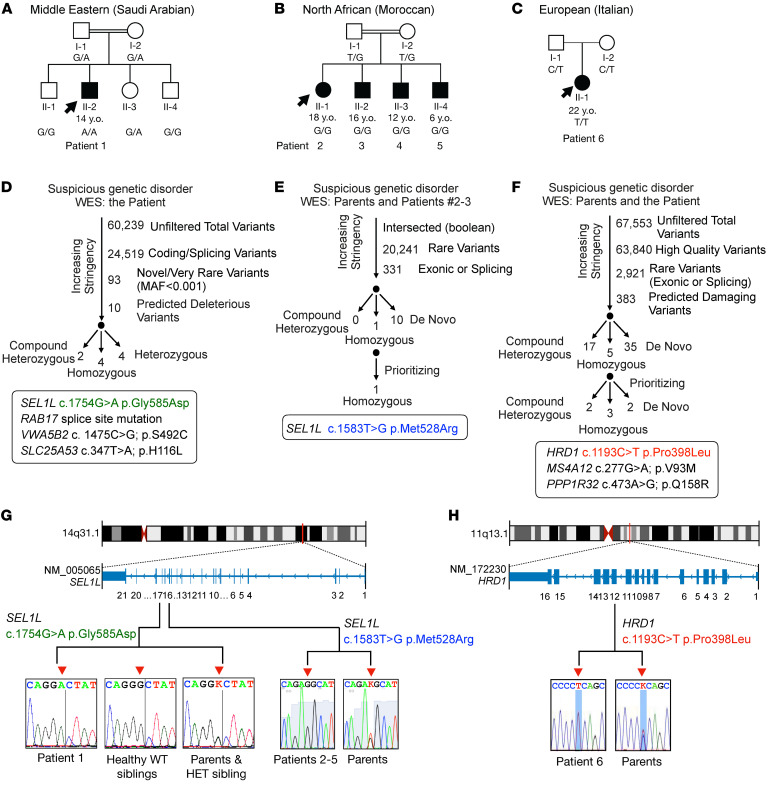
Genetic analysis pipeline and identification of bi-\allelic *SEL1L* and *HRD1* variants in patients. (**A**–**C**) Family pedigrees for the kindreds from Saudi Arabia (**A**, patient 1, consanguineous), Morocco (**B**, patients 2–5, consanguineous), and Italy (**C**, patient 6), showing autosomal recessive inheritance. Shaded shapes, individuals with symptoms; arrows, probands. Ages indicated are as of 2022. (**D**–**F**) Genetic analysis pipeline of WES data for (**D**) patient 1 (SEL1L p.G585D), (**E**) patients 2–3 (SEL1L p.M528R), and (**F**) patient 6 (HRD1 p.P398L). (**G** and **H**) Exonic and chromosomal location of the *SEL1L* (**G**) and *HRD1* (**H**) variants as well as Sanger sequencing confirmation of the patients and healthy family members from parts **A–C**. Red arrowheads, nucleotide changes; K, heterozygosity.

Six patients from 3 unrelated families in Saudi Arabia (patient 1), Morocco (patient 2–5), and Italy (patient 6) were suspected of inherited genetic disease during clinical visits (Figure 1, A–C). Patients 1, 2, 3, and 6 were subjected to WES of DNA samples (Figure 1, D–F). WES results were stringently filtered for novel variants by excluding variants with low sequencing quality, in the noncoding region, with high frequency in the population, or likely to be benign in silico (Figure 1, D–F). We failed to identify any known variants linked to inherited neurological or metabolic disorders, but rather noted 3 variants linked to the same protein complex/pathway, namely SEL1L-HRD1 ERAD: *SEL1L* p.Gly585Asp (NM_005065: exon 17: c.1754G>A) in the Saudi Arabian patient (patient 1), *SEL1L* p.Met528Arg (exon 16: c.1583T>G) in the Moroccan patients (patient 2 and 3), and *HRD1* p.Pro398Leu (NM_172230: exon 12: c.1193C>T) in the Italian patient (patient 6) (Figure 1, D–F, and Table 1). Indeed, homozygous *SEL1L* p.Met528Arg was the only variant shared between patients 2 and 3, but not found in the healthy parents. Although additional variants including homozygous, heterozygous, compound heterozygous, and de novo mutations were identified in the Saudi Arabian (patient 1) and Italian (patient 6) patients (Figure 1, D and F, and Supplemental Table 1; supplemental material available online with this article; https://doi.org/10.1172/JCI170054DS1), *SEL1L* p.Gly585Asp and *HRD1* p.Pro398Leu variants were considered as potential candidates based on their biological relevance as reported in mice (2, 3, 5) and also according to the American College of Medical Genetics (ACMG) and the Association for Molecular Pathology (AMP) 2015 guidelines for clinical interpretation of genetic variants (52). Indeed, neither of the SEL1L variants was found in public genetic variants databases, such as 1000gp3 (https://www.internationalgenome.org/), ESP6500 (https://esp.gs.washington.edu/drupal/), ExAC (https://gnomad.broadinstitute.org/), and gnomAD (https://gnomad.broadinstitute.org/), and both variants were consistently predicted to be damaging using various prediction tools, such as Combined Annotation Dependent Depletion (CADD) (https://cadd.gs.washington.edu/), PolyPhen2-HVAR (http://genetics.bwh.harvard.edu/pph2/), Sorting Intolerant from Tolerant (SIFT) (https://sift.bii.a-star.edu.sg/), LIST-S2 (https://list-s2.msl.ubc.ca/?session=838295C8507C38640C29677123B49248), Mendelian Clinically Applicable Pathogenicity (M-CAP) (http://bejerano.stanford.edu/mcap/), BayesDel addAF (https://fenglab.chpc.utah.edu/BayesDel/BayesDel.html), DEOGEN2 (http://babylone.3bio.ulb.ac.be/MutaFrame/), Functional Analysis through Hidden Markov Models (FATHMM-MKL) (http://fathmm.biocompute.org.uk/), MutationAssessor (http://mutationassessor.org/r3/), MutationTaster (https://www.mutationtaster.org/), and PrimateAI (https://github.com/Illumina/PrimateAI) (Table 1). Similarly, allele frequency of HRD1 p.Pro398Leu was low in the population and was predicted to be benign to damaging in various prediction databases (Table 1). *SEL1L* and *HRD1* genes are located on chromosomes 14 and 11, respectively (Figure 1, G and H). Using Sanger sequencing, we confirmed that the 3 variants were found to be homozygous in all patients, but heterozygous in all parents (Figure 1, G and H). Three siblings for patient 1 were either heterozygous or WT for the SEL1L allele (Figure 1, G and H).

### Clinical features of the patients.

**Figure 2:**
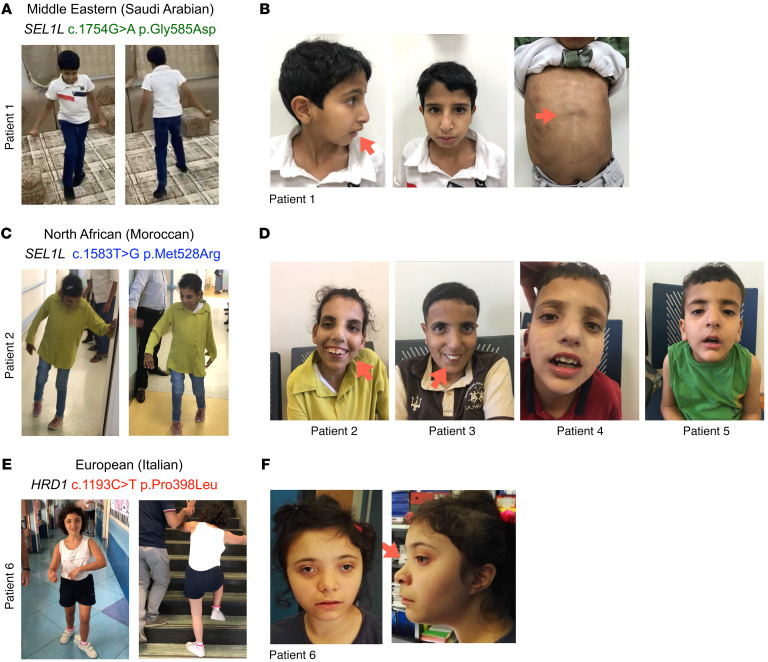
Clinical features of patients carrying *SEL1L* and *HRD1* variants. (**A**, **C**, **E**) Photos of patient 1 (**A**), patient 2 (**C**), and patient 6 (**E**) showing clumsy and wide gait, with need of external supports in **C** and **E**. (**B**, **D**, and **F**) Photos of the patients showing dysmorphisms (red arrows). (**B**) Patient 1 at 13 years (left, middle) and 11 years (right) of age, showing overbite (left, middle), downslanting palpebral fissures, and pectus excavatum (right). (**D**) Patients 2–5 at ages of 17 (patient 2), 15 (patient 3), 11 (patient 4), and 5 years (patient 5), showing overbite (patients 2 and 3) and downslanting palpebral fissures (patient 2–5). (**F**) Patient 6 at 16 years old, showing hypertelorism and flat nasal bridge (right).

Patient 1, a 14-year-old boy born to healthy consanguineous Saudi Arabian parents (with 3 healthy siblings) showed hypotonia (poor sucking and floppiness) and microcephaly at 4 months of age. Three siblings, 2 WT and 1 heterozygous at the *SEL1L* G585 locus, were healthy. The patient presented with global developmental delay (sat at 2 years of age, walked at 5, uttered mama/dada at 6, and is not at this writing toilet trained), moderate-severe intellectual disability (limited 2-word sentences and cannot count to 3, with an IQ of 35), and central hypotonia (with brisk deep tendon reflexes, wide-based gait with hands held on the side to support his balance) (Figure 2A, Table 1, Supplemental Table 2, and Supplemental Video 1). Physical examination performed at the age of 13 years revealed that he was underweight (–3.9 SD), and showed short stature (–3.9 SD), microcephaly (–4.2 SD), subtle facial dysmorphism (downslanting palpebral fissures and overbite), pectus excavatum (Figure 2B), a moderate degree of joint hyperlaxity, and shawl scrotum. The patient had a total of 3 seizures at 8 years of age with largely normal electroencephalogram (EEG). His medical history was notable for frequent airway infections, although his workup did not suggest immunodeficiency. Other than hypotonia, neurological examination was largely normal.

Patients 2, 3, 4, and 5, 4 Moroccan siblings, 1 female (the proband, born 2005) and 3 males (born 2007, 2011 and 2017), born to healthy consanguineous parents (Figure 1B), presented since a few months of age with developmental delay, intellectual disability, speech delay, short stature, seizures, and ataxic gait (progressive with age). The 2 older siblings (patients 2 and 3) had single seizure history, and the 2 younger siblings (patients 4 and 5) showed microcephaly. The proband showed severe spastic and ataxic gait, falls, wide-based gait, pes cavus and equinus, mild dystonia, paraparesis with pyramidal signs of lower limbs, with brisk, diffused tendon reflexes and clonus, pyramidal extension of the first toe, and bilateral positive Babinski sign (Figure 2C, Table 1, Supplemental Table 2, and Supplemental Video 2). The proband showed varus equus, scoliosis, and arched palate. The 4 patients also shared facial dysmorphism, including downslanting palpebral fissures and overbite (Figure 2D), and were diagnosed with unilateral maculopathy, pallor of temporal poles, and severe corneal dystrophy. MRI of the proband showed small cavities in the frontal periventricular area with nonspecific ventricular dilatation (Supplemental Figure 1). EEG of the proband showed generalized discharges of polyspikes and slow waves.

Patient 6, an Italian girl born to healthy nonconsanguineous parents in 2001, presented since her first months of age with hypotonia and severe drug-resistant seizures that were resolved by the age of 14 years (Figure 1C). She exhibited intellectual disability, speech delay, stereotypic movements, a clumsy gait (Figure 2E, Table 1, Supplemental Table 2, and Supplemental Video 3), and dysmorphic facial features (Figure 2F). Physical examination at 16 years of age revealed that she was underweight (37.5 kg body weight, <5th percentile, *z* score = –3.45), and showed short stature (height 139.5 cm, <5th percentile, *z* score = –3.9), and microcephaly (head circumference 51.8 cm, <5th percentile, *z* score = –2.6). Brain MRI performed in the first year of life revealed a cerebellar cyst without other notable findings. When repeated at the age of 21 years, an abnormal signal was detected in the globus pallidum and substantia nigra (not shown). Cardiac and abdomen ultrasounds were both normal. The patient has been on risperidone (Table 1) since the COVID-19 outbreak because of worsening of her behavior with agitation and aggression.

In summary, all 6 patients were symptomatic at infancy and presented with neurodevelopmental disorder, developmental delay, intellectual disability, and facial dysmorphisms (Table 1 and Supplemental Table 2). Four out of six patients had microcephaly. Two patients showed hypotonia, with floppiness, unsteady and clumsy gait, and difficulty in walking, but no frank ataxia, while the other 4 patients from the Moroccan family exhibited severe ataxia that progressed with age. Interestingly, hypotonia in the 2 patients did not progress with age, and in fact the Italian patient was no longer hypotonic at the most recent evaluation in June 2022. No notable abnormalities were observed in routine blood chemistry tests (glucose, electrolytes, blood urea nitrogen, creatinine, aspartate aminotransaminase, alanine aminotransaminase, and albumin) and complete blood counts in all 6 patients.

### SEL1L and HRD1 variants are hypomorphic with impaired ERAD function.

**Figure 3:**
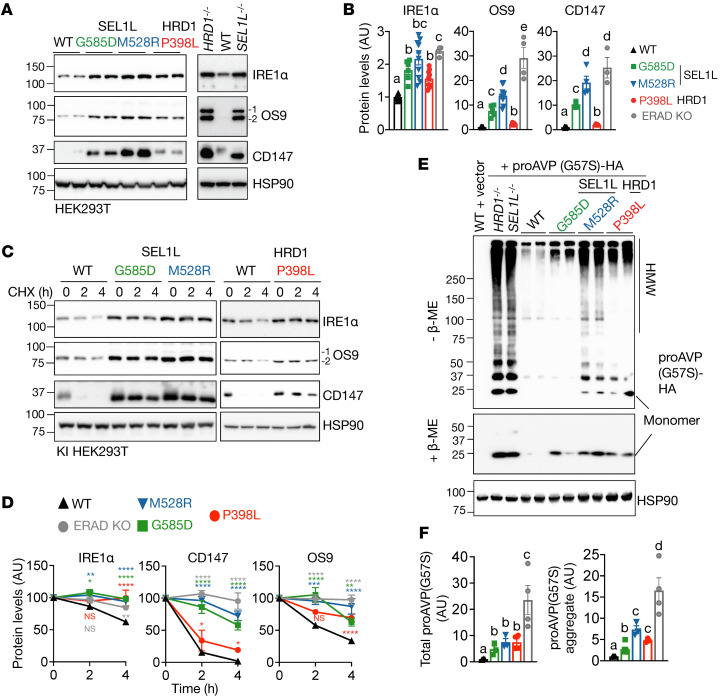
*SEL1L* and *HRD1* variants are hypomorphic with impaired ERAD function toward misfolded endogenous and model substrates. (**A** and **B**) Western blot analysis of known ERAD endogenous substrates IRE1α, OS9, and CD147 in KI HEK293T cells, with quantitation shown in **B**. *n* = 4–16 (IRE1α); *n* = 4–12 (OS9); *n* = 3–12 (CD147). OS9.1 and OS9.2 were quantified together as OS9. *SEL1L^–/–^* and *HRD1^–/–^* HEK293T cells were included as controls. (**C** and **D**) Cycloheximide (CHX) chase analysis of known ERAD endogenous substrates IRE1α, OS9, and CD147 in KI HEK293T cells, with quantitation shown in **D**. *n* = 3–8 (IRE1α); *n* = 3–6 (OS9); *n* = 3–8 (CD147). SEL1L and HRD1 variants were analyzed separately with their own WT controls. Quantitation normalized to WT controls. Western blot data for ERAD KO samples shown in Supplemental Figure 3. (**E** and **F**) Reducing and nonreducing SDS-PAGE and Western blot analysis of HMW aggregates of proAVP(G57S) in KI HEK293T cells, with quantitation shown in **F**. *n* = 3–6 for group. *n*, individual cell samples. For **A**, **C**, and **E**, *SEL1L* and *HRD1*-KO HEK293T cells were included as controls and quantitated as ERAD KO. The replicates in Western blot are technical replicates. Data are represented as means ± SEM. Quantitation of the band intensities was compared using 1-way ANOVA with post hoc Tukey-Kramer test (**B** and **F**) or 2-way ANOVA followed by multiple comparisons test (**D**). For **B**, comparisons between different letters (a–d) represents *P* < 0.05. *P < 0.05; **P < 0.01; ***P < 0.001; ****P < 0.0001.

To investigate whether and how these disease variants affect SEL1L-HRD1 ERAD function, we generated knockin (KI) HEK293T cells carrying the biallelic variants using the CRISPR/Cas9 system and verified by Sanger sequencing (Supplemental Figure 2, A–E). We then tested to determine whether these disease variants affect ERAD function by measuring protein stability and levels of known endogenous ERAD substrates such as inositol-requiring enzyme 1α (IRE1α) (41), OS9 (53), and cluster of differentiation 147 (CD147) (54) as well as the disease mutant of proarginine vasopressin (proAVP) Gly57Ser (Gly-to-Ser at residue 57) (47). Indeed, endogenous substrates became accumulated in all 3 KI HEK293T cell lines (Figure 3, A and B; see complete unedited blots in the supplemental material) due to protein stabilization, similar to that in SEL1L- or HRD1-KO HEK293T cells (Figure 3, C and D, and Supplemental Figure 3). Similarly, proAVP (Gly57Ser) accumulated in transfected KI cells, forming much more extensive high molecular weight (HMW) aggregates than those in WT cells (Figure 3, E and F).

A couple of points are worth noting here: first, although all variants caused substrate accumulation, the extent of substrate accumulation differed among the variants, the highest being the *SEL1L* M528R variant and the lowest being either the *SEL1L* G585D or *HRD1* P398L variant, which is consistent for both endogenous and model substrates (Figure 3, B and F). Second, the extent of substrate accumulation and HMW aggregation in KI cells was modest compared with that in KO cells (Figure 3, B and F), pointing to the hypomorphic nature, rather than loss of function, of these variants. Taken together, these data suggest that 3 variants are hypomorphic with moderate to severe ERAD dysfunction.

### Lack of an overt unfolded protein response in KI cells.

Intriguingly, we did not observe an overt unfolded protein response (UPR) in these KI cells, as demonstrated by the lack of IRE1α phosphorylation and X-box–binding protein 1 (*XBP1*) mRNA splicing as well as phosphorylation of protein kinase R-like ER kinase (PERK) and eukaryotic initiation factor-2α (eIF2α) (Supplemental Figure 4, A–D). ER chaperones, such as immunoglobulin heavy chain–binding protein (BiP) and protein disulfide isomerase (PDI), were accumulated in KI HEK293T cells (Supplemental Figure 4, E and F). These data point to a cellular adaptive response in cells expressing these disease variants.

### Sequence and structural analyses of SEL1L and HRD1 variants.

**Figure 4:**
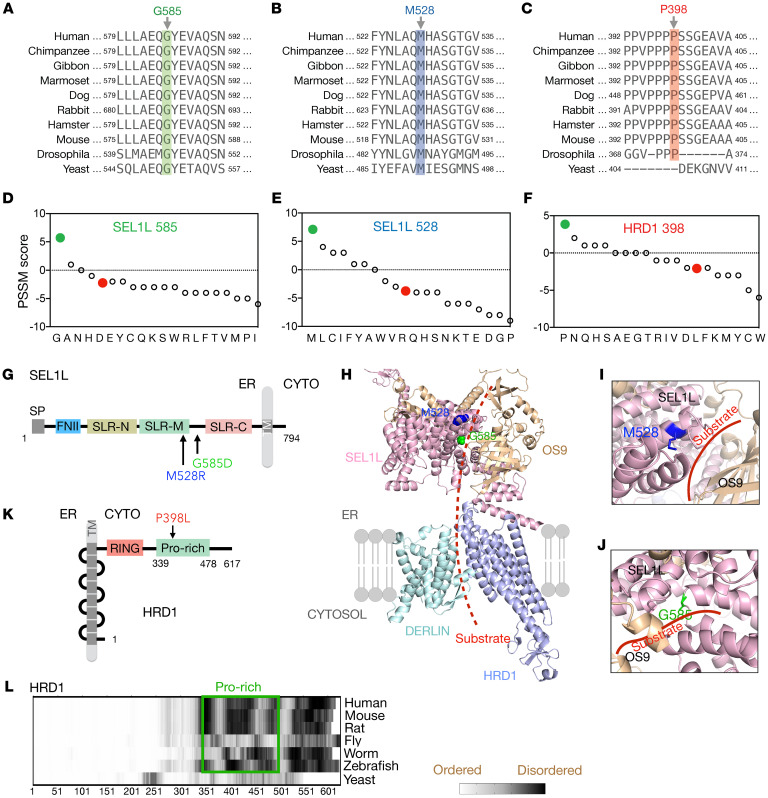
Sequence and structural analyses of *SEL1L* and *HRD1* variants. (**A**–**C**) The aa sequence alignment of SEL1L (**A** and **B**) and HRD1 (**C**) showing the conservation of residues across species. (**D**–**F**) PSSM scores for aa position in SEL1L (**D** and **E**) and HRD1 proteins (**F**), with WT in green and variants in red. (**G**–**K**) Schematic diagrams of human SEL1L (**G**) and HRD1 (**K**) with the location of the variants indicated. SP, signal peptide; FNII, fibronectin type II domain; SLR-N/M/C, Sel1-like repeats at N-terminal, middle-, and C-terminal; TM, transmembrane; CYTO, cytosol; RING, RING domain; Pro-rich, Proline-rich domain. (**H**–**J**) Structural prediction of human SEL1L/OS9/HRD1/DERLIN ERAD complex using AlphaFold2 with close-up views of SEL1L-M528 (blue) and G585 (green) areas shown in **I** and **J**. Red (dotted) line marks the putative substrate binding groove. (**L**) Comparison of disordered region of HRD1 across species, highlighting the disordered nature of the proline-rich domain.

We next asked how these variants affect ERAD function. We first performed in silico conservation and structural analyses. These variants affect conserved residues from yeast or drosophila to humans, with the exception of HRD1 Pro398, which is absent in yeast (Figure 4, A–C). Position-specific scoring matrix (PSSM) analysis (55) showed that all 3 variants replaced evolutionarily selected aa and that the mutations may be detrimental to SEL1L and HRD1 function (Figure 4, D–F). The *SEL1L* variants, p.Met528Arg and p.Gly585Asp, affect residues in the Sel1-like repeat–middle (SLR-M) and the linker region between SLR-M and -C, respectively (Figure 4G). To visualize these variants in the ERAD complex, we performed AI-based AlphaFold2 prediction network analysis (56) to model the structure of the human SEL1L (107–723 aa)-HRD1 (1–334 aa)-OS9 (33–655 aa)-DERLIN1 (1–213 aa) protein complex (Figure 4H). Structural modeling of the human SEL1L-HRD1 complex showed a great similarity to the cryogenic electron microscopy (Cryo-EM) structure of the yeast Hrd1p-Hrd3p complex (PDB ID 6VJZ) (12) (Supplemental Figure 5A). ConSurf conservation analysis (57) of SEL1L confirmed that both Met528 and Gly585 residues were in highly conserved patches (Supplemental Figure 5B).

SEL1L Met528 is predicted to be a part of the α-helix facing outward from the putative substrate binding groove and OS9 (Figure 4I). Mutation of Met528 to Arg is expected to seriously disrupt the α-helical structure and destabilize the protein. On the other hand, Gly585 is located on a loop between the 2 helices in the putative substrate-binding groove (Figure 4J). While mutation of Gly585 to Asp is not predicted to disrupt the α-helical structure, it is located in the substrate-binding groove in close proximity to the substrate(s) and lectins (OS9 and ERLEC1). Moreover, Pro398 of HRD1 is located in the proline-rich region (~50 Pro in a stretch of 140 aa) of its cytosolic domain, C-terminal to the really interesting new gene–finger (RING-finger) domain (Figure 4K). This proline-rich region is disordered based on IUPred2 prediction (58) (Figure 4L), with no predictable structure.

### SEL1L and HRD1 variants impair ERAD function via distinct mechanisms.

**Figure 5:**
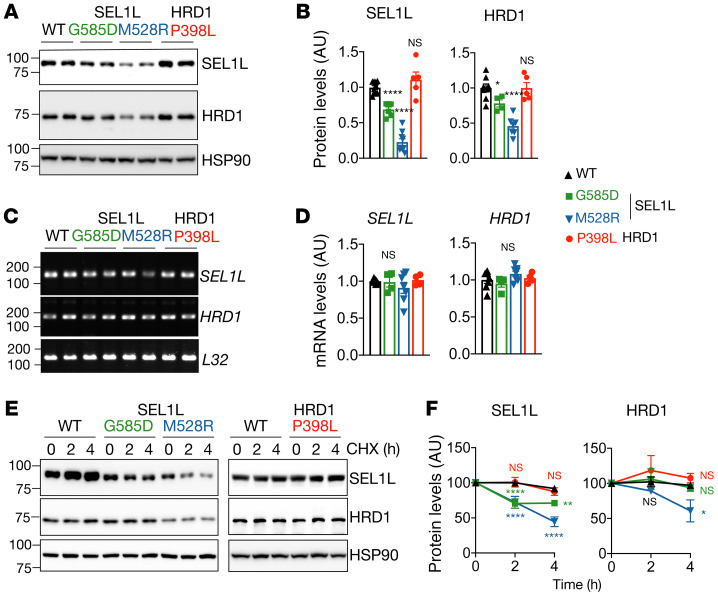
Reduced SEL1L-HRD1 protein level and stability for *SEL1L* M528R variant, not the other 2 variants. (**A** and **B**) Western blot analysis of SEL1L and HRD1 in KI HEK293T cells expressing indicated variants, with quantitation shown in **B**. *n* = 8–10 (WT); *n* = 4–6 (G585D); *n* = 7–9 (M528R). (**C** and **D**) RT-PCR analysis of *SEL1L* and *HRD1* transcript levels in KI HEK293T cells, with quantitation shown in **D**. *n* = 4–7 per group. *L32*, loading control. (**E** and **F**) Cycloheximide chase analysis of SEL1L and HRD1 in KI HEK293T cells, with quantitation shown in **F**. *n* = 10 (WT); *n* = 3–4 (G585D); *n* = 4–5 (M528R); *n* = 3–5 (P398L). SEL1L and HRD1 variants were analyzed separately with their own WT controls. Quantitation normalized to WT controls. *n*, individual cell samples. Data are represented as means ± SEM. *P < 0.05; **P < 0.01; ****P < 0.0001, 1-way ANOVA followed by Dunnett’s multiple-comparisons test (**B** and **D**); 2-way ANOVA followed by multiple comparisons test (**F**).

We next explored how these variants cause ERAD defects using KI HEK293T cells. We first measured protein levels of the SEL1L-HRD1 ERAD complex. Noticeably, *SEL1L^M528R^* KI HEK293T exhibited reduced SEL1L and HRD1 protein levels, by approximately 80% and 60%, respectively (Figure 5, A and B), uncoupled from their gene transcription (Figure 5, C and D). Indeed, both SEL1L and HRD1 proteins were unstable in *SEL1L^M528R^* KI cells (Figure 5, E and F). In contrast, *SEL1L^G585D^* exhibited a modest reduction of SEL1L and HRD1 protein levels, by approximately 20% to 30% (Figure 5, A and B), without changes in mRNA levels (Figure 5, C and D). *SEL1L^G585D^* had a subtle effect on the stability of SEL1L protein, but not HRD1 protein, in KI HEK293T cells (Figure 5, E and F). This reduction in ERAD protein levels was unlikely to explain the ERAD defects associated with *SEL1L^G585D^*-expressing cells, as heterozygosity of SEL1L or HRD1 is sufficient for ERAD function (20, 34, 43, 44). On the other hand, *HRD1^P398L^* had no effect on either protein levels or stability of SEL1L and HRD1 (Figure 5, A, B, E, and F). Hence, the *SEL1L^M528R^* variant causes ERAD dysfunction by reducing protein stability and levels of the SEL1L-HRD1 complex, but not *SEL1L^G585D^* and *HRD1^P398L^*.

**Figure 6:**
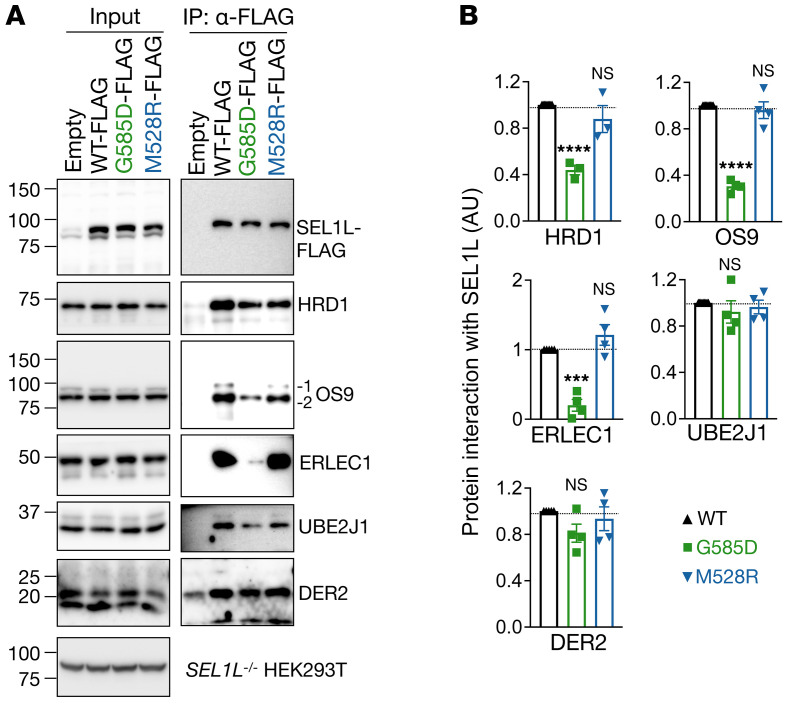
*SEL1L G585D* variant, but not M528R, impairs substrate recruitment. (**A** and **B**) IP of FLAG-agarose in *SEL1L^–/–^* HEK293T cells transfected with G585D or M528R SEL1L-FLAG to test their interactions with components of the ERAD complex, with quantitation shown in **B**. *n* = 3–4 per group. *n*, individual cell samples. Data are represented as means ± SEM. ***P < 0.001; ****P < 0.0001, 1-way ANOVA followed by Dunnett’s multiple-comparisons test (**B**).

Given the location of SEL1L G585 residue, we next asked whether *SEL1L^G585D^* affects substrate recruitment. During ERAD, substrates are recruited by lectins such as OS9 and ERLEC1 to the SEL1L-HRD1 complex, which also includes ubiquitin-conjugating E2 enzyme J1 (UBE2J1) and DERLIN proteins (18). To circumvent the confounding issue of reduced SEL1L and HRD1 protein levels in SEL1L KI cells, we used an overexpression system in *SEL1L^–/–^* HEK293T cells. Surprisingly, the *SEL1L^G585D^* variant significantly reduced its interactions with 2 lectin proteins (ERLEC1 and OS9), by approximately 70% to 80%, and with HRD1 by approximately 50% compared with that of WT SEL1L (Figure 6, A and B). In contrast, SEL1L interaction with UBE2J1 and DERLIN2 was not affected in *SEL1L^G585D^*-expressing cells (Figure 6, A and B). In contrast, *SEL1L^M528R^* did not affect the interaction between SEL1L and HRD1 or other ERAD components in transfected *SEL1L^–/–^* HEK293T cells (Figure 6, A and B). Hence, unlike *SEL1L^M528R^*, the *SEL1L^G585D^* variant impairs ERAD function by attenuating substrate recruitment. This conclusion is in line with the prediction that SEL1L G585 faces the substrate-binding groove and is in close proximity to OS9 (Figure 4J).

**Figure 7:**
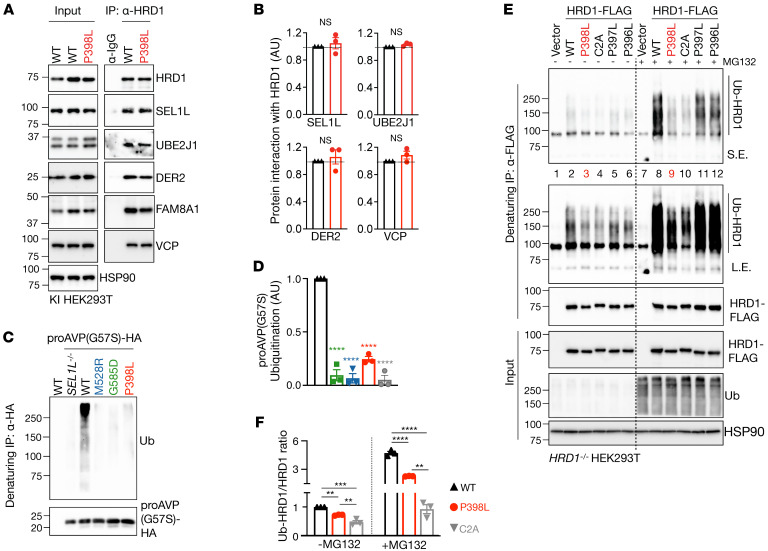
*HRD1* P398L variant impairs HRD1 ubiquitination. (**A** and **B**) IP of HRD1 in *HRD1^P398L^* KI HEK293T cells to test its interaction with components of the ERAD complex, with quantitation shown in **B**. *n* = 3 per group. (**C** and **D**) Denaturing IP of HA-agarose in KI HEK293T cells expressing indicated variants transfected with a model substrate proAVP (G57S)-HA to measure substrate ubiquitination, with quantitation shown in **D**. *n* = 3 per group. (**E** and **F**) Denaturing IP of FLAG-agarose in *HRD1^–/–^* HEK293T cells transfected with indicated HRD1 variants, with or without 10 μM MG132 for 2 hours, to measure HRD1 ubiquitination, with quantitation shown in **F**. *n* = 3 per group. *n*, individual cell samples. Data are represented as means ± SEM. **P < 0.01; ***P < 0.001; ****P < 0.0001, 2-tailed Student’s *t* test (**B**); 1-way ANOVA followed by Dunnett’s multiple-comparisons test (**D** and **F**).

In *HRD1^P398L^* KI HEK293T cells, the interactions of HRD1 with other ERAD components, such as SEL1L, UBE2J1, DER2, valosin-containing protein (p97/VCP), and family with sequence similarity 8 member A1 (FAM8A1), were comparable to those in WT HEK293T cells (Figure 7, A and B). However, substrate ubiquitination was significantly attenuated in *HRD1^P398L^* KI HEK293T cells, similar to that in the other 2 SEL1L variants (Figure 7, C and D), providing further support for ERAD dysfunction. Given that HRD1 P398L is close to the RING domain (Figure 4K), we next asked whether HRD1 P398L may affect HRD1 activity by modulating its ubiquitination using denaturing immunoprecipitation (IP) followed by Western blot. For unknown reasons, we failed to detect ubiquitination of endogenous HRD1 proteins in WT and KI cells even with the treatment of MG132 (Supplemental Figure 6). Upon transfection in *HRD1^–/–^* HEK293T cells, P398L mutation attenuated HRD1 ubiquitination compared with those in WT cells (lanes 3 and 9 versus 2 and 8, Figure 7, E and F). The effect of P398L on HRD1 ubiquitination was similar to that of HRD1 C2A mutation (lanes 4 and 10), a mutation in the RING domain known to abolish HRD1 E3 activity (8). Interestingly, mutation of the neighboring HRD1 Pro to Leu (P397L or P396L) had a much milder effect on HRD1 ubiquitination (lanes 5–6 and 11–12, Figure 7, E and F). MG132 treatment enhanced HRD1 ubiquitination in all samples (lanes 8–12 versus 2–6, Figure 7, E and F), suggesting that HRD1 ubiquitination may contribute to its turnover. These data suggest that HRD1 P398L affects HRD1 ubiquitination, which may contribute to its dysfunction. Taking these data together, we conclude that these 3 variants cause ERAD dysfunction at distinct steps of ERAD, including substrate recruitment (*SEL1L^G585D^*), SEL1L-HRD1 protein stability and complex formation (*SEL1L^M528R^*), and HRD1 activity (*HRD1^P398L^*).

## Discussion

**Figure 8:**
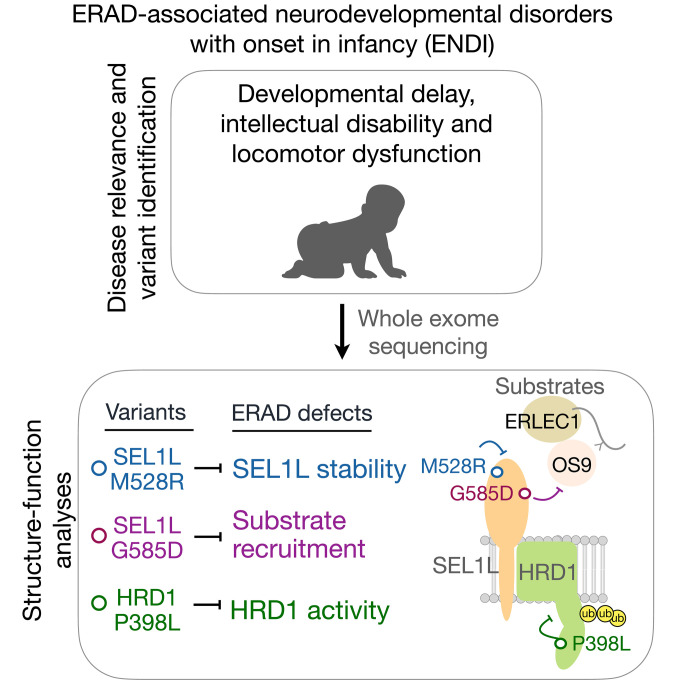
Our model for disease-causing SEL1L-HRD1 hypomorphic variants in ENDI. Human ENDI variants identified in patients through WES are hypomorphic and cause a partial loss of function of SEL1L-HRD1 ERAD via distinct mechanisms such as SEL1L protein and ERAD complex stability (*SEL1L^M528R^*), substrate recruitment (*SEL1L^G585D^*), and HRD1 activity (*HRD1^P398L^*).

This study reports 3 variants in *SEL1L* and *HRD1* genes in 6 patients from 3 unrelated families. These patients manifest similar clinical features, including developmental delay, microcephaly, intellectual disability, facial dysmorphism, hypotonia, and ataxia. Using KI HEK293T cells expressing individual variants, we further show that these variants impair ERAD function at distinct steps of ERAD, including substrate recruitment, SEL1L-HRD1 protein stability and complex formation, and HRD1 activity (Figure 8). We speculate that the phenotypic variations among these patients may reflect different levels of ERAD dysfunction associated with the variants and/or less likely, possible effects of other non-ERAD variants.

A few additional variants were identified from the Saudi Arabian (patient 1) and Italian (patient 6) patients (Supplemental Table 1). Most of the heterozygous and compound heterozygous variants were predicted to be benign by the pLI score (the intolerance of the gene to loss of function) and variant effect prediction tools (CADD, PolyPhen-2 HVAR, SIFT), except for the heterozygous Furry-like (FRY-like) transcription coactivator (*FRYL)* variant (*FRLY* c.7490C>G, p.T2497R) identified in patient 1; however, *Fryl* heterozygous mutant mice were found to be normal compared with WT littermates, while homozygous mutant mice showed lower birth rate and renal defects (hydronephrosis) if they survived (59), suggesting that the *FRYL* variant may not be disease relevant in patient 1. Similarly, although several additional homozygous variants were identified, the reported functions of these proteins are not biologically relevant to the symptoms observed in our patients, e.g., Ras-associated protein rab17 (*RAB17),* which encodes a GTPase to enable GDP-binding activity (60), von Willebrand factor A containing 5B2 domain (*VWA5BA*), which belongs to the family of von Willebrand factors crucial for primary platelet and collagen adhesion function (61), and Solute carrier family 25, member 53 (*SLC25A53*), which is predicted to be an integral component of the mitochondrial inner membrane (Alliance of Genome Resources, https://www.alliancegenome.org/) with unknown function. Among them, although mutations of other RAB family proteins have been linked to neurological disorders (62), RAB17 is an epithelial cell–specific GTPase (63) and is expressed at a very low level in the central nervous system (GTExPortal, gtexportal.org). Similarly, 2 other variants identified in patient 6, membrane-spanning 4-domains, subfamily a, member 12 (*MS4A12*) and protein phosphatase 1 regulatory subunit 32 (*PPP1R32*), are associated with colon cancer (64, 65) and ciliary movements (66), respectively. Given the (patho-)physiological importance of SEL1L-HRD1 ERAD (2, 3, 5, 67) and given that the M528R variant is the only variant shared among the affected siblings from the Moroccan family, we believe that SEL1L-HRD1 ERAD variants are most likely to be disease causing in these patients.

Comparing disease-variant KI HEK293T cells to ERAD KO HEK293T cells, our biochemical analyses showed that these variants attenuated ERAD function. Since all the parents and some healthy siblings were heterozygous for the variant, we propose that all these variants cause more than a 50% reduction in ERAD function. Further comparisons among the 3 variants showed that SEL1L M528R may be the most severe one. This may account for the differences in clinical features between patient 2 to 5 with the *SEL1L^M528R^* variant (ataxia and microcephaly) and the other 2 patients, 1 and 6, with the *SEL1L^G585D^* and *HRD1^P398L^* variants (hypotonia). While the underlying molecular mechanisms are distinct for these variants in causing ERAD dysfunction, they all invariably cause ERAD dysfunction, leading to the stabilization and accumulation of endogenous ERAD substrates. Hence, these studies suggest that there is a threshold requirement for SEL1L-HRD1 ERAD function essential for normal neuronal function in humans.

We reported that these human SEL1L-HRD1 variants compromised ERAD via distinct mechanisms. Specifically, in HRD1 P398L KI HEK293T cells, HRD1 ERAD function was impaired. Following overexpression in HRD1-deficient HEK293T cells, we found that the HRD1 P398L variant impaired HRD1 ubiquitination. While this finding is potentially interesting, as it may reflect HRD1 autoubiquitination as reported by Baldridge et al. (28), we are aware that overexpression of HRD1 likely alters the stoichiometric ratios of the ERAD components that do not accurately reflect those of the endogenous HRD1 complex. The “ubiquitinated HRD1” result from (partially) unassembled and misfolded HRD1 that are targeted for proteasome-dependent degradation. Studies are underway to explore whether HRD1 P398L affects autoubiquitination of the RING domain specifically related to channel gating or other lysine residues.

In the accompanying paper (68), we reported an additional 5 patients carrying another *SEL1L* variant (*SEL1L* p.Cys141Tyr) identified from a Slovakian Romani family. This group of patients exhibited not only similar neurological disorders, but severe agammaglobulinemia resulting from the lack of B cells. This difference in clinical manifestation is likely due to the fact that SEL1L p.Cys141Tyr is the most severe variant among the four. Moreover, a *SEL1L* mutation (*p.Ser658Pro)* was previously identified in Finnish hounds with cerebellar ataxia (also known as cerebellar ataxia Finnish hound type [CAFH]) (69), further suggesting that *SEL1L* may play an important role in maintaining normal neurological function or neurodevelopment. These findings provide strong experimental support for the notion that hypomorphic SEL1L-HRD1 variants are pathogenic in humans. With that said, how these variants are linked to neurological defects in these patients remains to be investigated and is of great interest going forward. Although this lacks substantial evidence in humans, we speculate that SEL1L-HRD1 ERAD variants cause disease via substrate-dependent and cell-type–specific manners, as none is associated with an overt UPR. Other mechanisms, such as organellar dysfunction, may also contribute to this pathological process.

This study reports what we believe is the first set of human patients carrying variants in the core components of a key protein degradative machinery, providing key evidence for its pathophysiological importance in humans. It is worth noting that several variants have been identified in p97/VCP, another key component of the ERAD machinery involved in protein retrotranslocation from the ER. However, unlike SEL1L-HRD1 ERAD variants, these p97/VCP variants cause multisystem disorders (70–72). Differences in clinical features between these 2 sets of patients are likely due to the fact that, in addition to ERAD, p97/VCP is involved in a wide variety of other cellular functions, including genomic stability, translational stress response, and RNA biology (71). Moreover, a number of variants in genes involved in protein glycosylation have been reported to cause congenital disorders of glycosylation (CDG), manifestations of which also largely include neurodevelopmental delay and variable facial dysmorphism (73–78) — clinical consequences similar to those of our ENDI patients described in this study. This similarity is not surprising, as glycosylation is intimately associated with ER protein folding, maturation, and degradation (79). However, CDG patients also exhibit multisystemic symptoms, including hypoglycemia and liver, skin, gastrointestinal, and coagulation abnormalities (75), which were not observed in ENDI patients. Hence, identifying Mendelian disorders caused by mutations in core ERAD components is essential in delineating the importance of ERAD in humans.

ENDI is a rare neurodevelopmental disorder associated with SEL1L-HRD1 ERAD and characterized by infantile-onset developmental delay, intellectual disability, microcephaly, facial dysmorphisms, hypotonia, and/or ataxia. Intellectual disability affects about 1% to 3% of the population (80, 81), while ataxia has an estimated overall prevalence of 26 in 100,000 in children (82). Our data suggest that evaluating SEL1L-HRD1 ERAD has diagnostic values for those with intellectual disability, developmental delay, and ataxia. While it is currently rare, we expect that more SEL1L-HRD1 ERAD variants will surface as evidence grows for its importance in humans. Options for treating patients with ENDI are currently very limited, but this study provides a framework for our future effort to target this important ERAD complex.

## Methods

### Human subjects.

Six patients from 3 families were identified and included in the study. The patient cases were gathered through the web-based tool GeneMatcher (83) (https://genematcher.org/statistics/). The Saudi Arabian boy was born in 10/2009 to a gravida 2, para 1, abortion 0 29-year-old healthy mother and a 29-year-old father following an uneventful full-term pregnancy and spontaneous vaginal delivery. He presented with global developmental delay, intellectual disability, and hypotonia. MRI at 4 months of age suggested nonspecific periventricular white matter signal. The patient was officially diagnosed with short stature at 5 years of age and has shown limited response to growth-hormone therapy. The patient was diagnosed with cataract at the age of 6 and was treated with lens extraction and intraocular lens implant placement. The patient was diagnosed with hypothyroidism and has been on 25 mcg of l-thyroxine because of elevated thyroid-stimulating hormone (TSH). Other than hypotonia, neurological examination was largely normal. The last doctor visit was in May 2022.

Four Moroccan siblings, 1 female (born in 06/2005) and 3 males (born in 12/2007, 02/2011, and 10/2017) displayed developmental delay, intellectual disability, speech delay, short stature, seizures, and ataxic gait (progressive with age). Brain MRIs of patient 2 (Moroccan family proband), aged 14 years, showed small cavities in the frontal periventricular area with nonspecific ventricular dilatation on coronal T2, coronal FLAIR, and axial T2 weighted images and thin corpus callosum with no anomalies of basal ganglia or at the infratentorial level of sagittal plane. Blood tests suggested vitamin D deficiency and an infection at the time of tests on 06/2022. Family history was notable for parents being first cousins. No similarly affected relatives were found in the family. The last doctor visit was in 06/2022.

The Italian girl (born in 11/2001) presented with intellectual disability, speech delay, hypotonia, severe drug-resistant seizures, stereotypies, and dysmorphic features. The patient showed no autism spectrum disorder traits. The last doctor visit date was in June 2022.

### CRISPR/Cas9-based KO and KI HEK293T cells.

HEK293T cells, obtained from ATCC, were cultured at 37°C with 5% CO_2_ in DMEM with 10% fetal bovine serum (Fisher Scientific). To generate SEL1L- or HRD1-KO HEK293T cells, sgRNA oligonucleotides designed for human *SEL1L* (5′-GGCTGAACAGGGCTATGAAG-3′) and human *HRD1* (5′-GGACAAAGGCCTGGATGTAC-3′) were inserted into lentiCRISPR, version 2 (Addgene, 52961). Cells grown in 10 cm petri dishes were transfected with indicated plasmids using 5 μl of 1 mg/ml polyethylenimine (PEI) (MilliporeSigma) per 1 μg of plasmids for HEK293T cells. The cells were cultured 24 hours after transfection in medium containing 2 μg/ml puromycin for 24 hours and then in normal growth medium.

*SEL1L^M528R^, SEL1L^G585D^*, and *HRD1^P398L^* KI HEK293T cells were generated using the CRISPR/Cas9 Homology-Directed Repair (HDR) system (Integrated DNA Technologies [IDT]); 5 μL of 100 μM Alt-R crRNA (IDT) with gRNA sequence was mixed with 5 μL of 100 μM Alt-R tracrRNA (IDT) containing the Cas9 interacting sequence. To anneal the oligos, the duplex mixture was heated at 95°C for 5 minutes and then cooled at room temperature for 20 minutes, and 9 μL of the guide complex was incubated with 6 μL of the 62 μM Alt-R Cas9 enzyme (IDT) at room temperature for 20 minutes; 5 μL of the ribonucleoprotein (RNP) complex, together with 1.2 μL of the 100 μM HDR donor oligo (IDT) and 1.2 μL of the 100 μM Alt-R Cas9 electroporation enhancer (IDT), was added into the 100 μL HEK293T cell suspension (about 5 × 10^5^ cells) in electroporation solution (Ingenio). The mixture was transferred into a 0.2 cm cuvette, and electroporation was performed using Lonza Nucleofector IIb (Lonza). To prepare cell culture media, 3.4 μL pf 0.69 mM Alt-R HDR Enhancer V2 (IDT) was added to 2,000 μL DMEM with 10% fetal bovine serum (Fisher Scientific). After electroporation, cell suspension was added to the cell culture media, and the mixture was incubated in 4 wells of a 24-well plate (500 μL per well). The cells were cultured at 37°C with 5% CO_2_. After 5 days of incubation, the genomic DNA of the cell culture was extracted with 50 mM NaOH. DNA fragments covering the target sites were amplified by PCR using HotStart Taq 2× PCR Master Mix (ABclonal) and analyzed by Sanger Sequencing (Eurofins Genomics US) to estimate the percentage of mutant allele in the cell pool. In parallel, cells were diluted into 8 cells per mL and cultured in 96-well plates (100 μL per well) for single-cell isolation. After 10 days, 100 single-cell colonies were transferred into 24-well plates. The *SEL1L^M528^* region of each colony was amplified by a 50 μL PCR reaction, and 25 μL of the PCR product was treated with endonuclease NsiI (NEB) in rCutSmart Buffer (NEB), incubated at 37°C overnight. PCR products that were resistant to NsiI digestion were further analyzed by Sanger sequencing. The *SEL1L^G585D^* and the *HRD1^P398L^* regions were amplified using a 25 μL PCR reaction and sequenced. Cell colonies with homozygous *SEL1L^M528R^*, *SEL1L^G585D^*, or *HRD1^P398L^* alleles were transferred into a 6-well plate for further experiments.

Sequences were as follows: crRNA (guide sequence): *SEL1L^M528R^*: 5′-CTAGCTCAGATGCATGCCAG-3′, *SEL1L^G585D^*: 5′-TACCTCCTCCTGGCTGAACA-3′, *HRD1^P398L^*: 5′-CACAGCCTCTCCTGAGCTGG-3′; HDR donor oligo (mutation sites are underlined): *SEL1L^M528R^*: 5′-AATTTAGCTTCTCAGGGAGGCCATATCTTGGCTTTCTATAACCTAGCTCAGAGGCATGCCAGTGGCACCGGCGTGATGCGATCATGTCACACTGCAGTGGAG-3′, *SEL1L^G585D^*: 5′-GGCGATTACAATGCTGCAGTGATCCAGTACCTCCTCCTGGCTGAACAGGACTATGAAGTGGCACAAAGCAATGCAGCCTTTATTCTTGATCAGAGTAAGG-3′, *HRD1^P398L^*: 5′-TGGCCCCCCATGGGCCCCTTTCCACCTGTCCCGCCTCCCCTCAGCTCAGGAGAGGCTGTGGCTCCTCCATCCACCAGTGCAGG-3′; amplification PCR primers: *SEL1L^M528R^*: F: 5′-AATCTGTATCAGTGTGTTAGCTTGTATTA-3′, R: 5′-AGACTTTCCTGCTGGGCAA-3′; *SEL1L^G585D^*: F: 5′-AAACCTGTTGACTTCTAAAGAGTAAGTGAAAACTT-3′, R: 5′-AATGTCAAATCCATTTCTACAGTCAACTCG-3′; *HRD1^P398L^*: F: 5′-CAGTCAGTGTGACCAGTGCT-3′, R: 5′-CTCACCCCCAAGAAGAACCC-3′; and sequencing primers: *SEL1L^M528R^*: 5′-CTTACAGATGGCATTGGAGTCAAGAGA-3′, *SEL1L^G585D^*: 5′-CCCACCTCACACAGTTGTTTAAGAATGT-3′, *HRD1^P398L^*: 5′-CCTCCGTCTTCTCTCTGCAG-3′.

### Plasmids.

The following plasmids were used in the study (h denotes human genes; m denotes mouse genes): pcDNA3-h-proAVP(G57S)-HA (described previously, ref. 47); *mSel1L* cDNA (cloned from mouse liver cDNA and inserted into the pcDNA3 to generate pcDNA3-mSEL1L[WT]-FLAG). Point mutations of *SEL1L* in this study were generated using site-directed mutagenesis. The SEL1L-FLAG mutants G585D and M528R were generated using the plasmid pcDNA3-mSEL1L(WT)-FLAG as the template. All plasmids were validated by DNA-Seq. The mutagenesis primers were as follows: mSEL1L-FLAG-F: 5′-CGCGGATCCACCATGCAGGTCCGCGTCAGGCTGTCG-3′, R: 5′-CGCTCTAGACTATTTATCATCATCATCTTTATAATCTCCGCCCTGTGGTGGCTGCTGCTCTGG-3′. G585D-F: 5′-TGGCTGAGCAGGACTACGAGGTGGC-3′, R: 5′-GCCACCTCGTAGTCCTGCTCAGCCA-3′. M528R-F: 5′-CCTCGCACAGAGGCACGCCAGCGGC-3′, and R: 5′-GCCGCTGGCGTGCCTCTGTGCGAGG-3′. hHRD1 cDNA was cloned from pcDNA3-hHRD1(WT)-Myc-His (a gift from Y. Ye, National Institute of Diabetes and Digestive and Kidney Disease, Bethesda, Maryland, USA) and inserted into the pcDNA3 to generate pcDNA3-hHRD1(WT)-FLAG. Point mutations of HRD1 in this study were also generated using site-directed mutagenesis. The HRD1-FLAG mutants P398L, C2A(C291A/C294A), P397L, and P396L were generated using the plasmid pcDNA3-hHRD1(WT)-FLAG as the template. Sequences were as follows: hHRD1-FLAG-F: 5′-GGCGGTACCATGTTCCGCACGGCAGTGATGATG-3′, R: 5′-GGCGGATCCTCATTTATCATCATCATCTTTATAATCTCCGCCGTGGGCAACAGGAGACTC-3′; P398L-F: 5′-GTCCCGCCTCCCCTCAGCTCAGGAGAG-3′, R: 5′-CTCTCCTGAGCTGAGGGGAGGCGGGAC-3′; P397L-F: 5′-CCTGTCCCGCCTCTCCCCAGCTCAGGAG-3′, R: 5′-CTCCTGAGCTGGGGAGAGGCGGGACAGG-3′; P396L-F: 5′-CCACCTGTCCCGCTTCCCCCCAGCTC-3′, R: 5′-GAGCTGGGGGGAAGCGGGACAGGTGG-3′; C2A-F: 5′-ATGGACAATGTCGCCATCATCGCCCGAGAAGAGATG-3′, R: 5′-CATCTCTTCTCGGgcGATGATGgcGACATTGTCCAT-3′.

### Western blot and antibodies.

Cells were harvested and snap-frozen in liquid nitrogen. The proteins were extracted by sonication in NP-40 lysis buffer (50 mM Tris-HCl at pH 7.5, 150 mM NaCl, 1% NP-40, 1 mM EDTA) with protease inhibitor (MilliporeSigma), DTT (MilliporeSigma, 1 mM), and phosphatase inhibitor cocktail (MilliporeSigma). Lysates were incubated on ice for 30 minutes and centrifuged at 16,000*g* for 10 minutes. Supernatants were collected and analyzed for protein concentration using the Bio-Rad Protein Assay Dye (Bio-Rad); 20–50 μg of protein was denatured at 95°C for 5 minutes in 5× SDS sample buffer (250 mM Tris-HCl pH 6.8, 10% sodium dodecyl sulfate, 0.05% bromophenol blue, 50% glycerol, and 1.44 M β-mercaptoethanol). Protein was separated using SDS-PAGE or Phos-tag gel (as described previously, refs. 84, 85), followed by electrophoretic transfer to PVDF (Fisher Scientific) membrane. The blots were incubated in 2% BSA/TBST with primary antibodies overnight at 4°C: anti-HSP90 (Santa Cruz Biotechnology Inc., sc-13119, 1:5,000), anti-SEL1L (home-made, ref. 33; 1:10,000), anti-HRD1 (Proteintech, 13473-1, 1:2,000), anti-OS9 (Abcam, ab109510, 1:5,000), anti-CD147 (Proteintech, 11989-1, 1:3,000), anti-IRE1α (Cell Signaling Technology, 3294, 1:2,000), anti-ERLEC1 (Abcam, ab181166, 1:5,000), anti-UBE2J1 (Santa Cruz Biotechnology Inc., sc-377002, 1:3,000), anti-DER2 (gift from Chih-Chi Andrew Hu, Houston Methodist Hospital, Houston, Texas, USA, ref. 86, 1:1,000), anti-VCP (Proteintech, 10736-1, 1:3000), anti-FAM8A1 (Proteintech, 24746-1, 1:3000), anti-FLAG (MilliporeSigma, F1804, 1:1,000), anti-HA (MilliporeSigma, H3663, 1:5,000), anti-PERK (Cell Signaling Technology, 3192, 1:5000), anti-eIF2α (Cell Signaling Technology, 9722, 1:5000), anti–p-eIF2α (Cell Signaling Technology, 9721, 1:1,000), anti-GRP78 BiP (Abcam, ab21685, 1:5000), and anti-PDI (Enzo Life Sciences, ADI-SPA-890-D, 1:5000). Membranes were washed with TBST and incubated with secondary antibodies, either HRP conjugated (Bio-Rad, 1:10,000), anti-rabbit IgG TrueBlot HRP (Rockland, 18-8816-33, 1:500), or anti-mouse IgG TrueBlot-HRP (Rockland 18-8817-31, 1:500), at room temperature for 1 hour for ECL chemiluminescence detection system (Bio-Rad) development. Band intensity was determined using Image lab (Bio-Rad) software (verison 6.1).

### IP.

For SEL1L-FLAG and HRD1 IP, HEK293T cells transfected with the indicated plasmids or KI HEK293T cells were snap-frozen in liquid nitrogen and whole-cell lysate was prepared in the IP lysis buffer (150 mM NaCl, 0.2% Nonidet P-40 [NP40], 0.1% Triton X-100, 25 mM Tris-HCl pH 7.5) at 4°C, supplemented with protease inhibitors, protein phosphatase inhibitors, and 10 mM *N*-ethylmaleimide. A total of approximately 5 mg protein lysates were incubated with 15 μl anti-FLAG agarose (MilliporeSigma, A2220) or 2 μl anti-HRD1 antibody (Proteintech, 13473-1) overnight at 4°C with gentle rocking. HRD1 IP lysates were incubated with 10 μl protein A agarose (Invitrogen, 20333) at 4^o^C for 2 hours after incubation. Incubated agaroses were washed 3 times with the IP lysis buffer and eluted in the SDS sample buffer at 95^o^C for 5 minutes followed by SDS-PAGE and immunoblot.

### Denaturing IP for ubiquitination assay.

HEK293T cells were transfected with proAVP(G57S)-HA plasmids for 24 hours and then treated with 10 μM MG132 for 2 hours. The cells were snap-frozen in liquid nitrogen, and whole-cell lysate was prepared in the NP-40 lysis buffer (50 mM Tris-HCl at pH7.5, 150 mM NaCl, 1% NP-40, 1 mM EDTA) with 1% SDS and 5 mM DTT, denatured at 95°C for 10 minutes, and centrifuged at 16,000*g* for 10 minutes. Subsequently, supernatants were diluted 1:10 with NP-40 lysis buffer and incubated with 15 μl anti-HA agarose (Thermo Fisher,26182) overnight at 4°C with gentle rocking. The incubated agaroses were washed 3 times with the NP-40 lysis buffer and eluted in the SDS sample buffer at 95°C for 5 minutes, followed by SDS-PAGE and immunoblot.

### Chemical treatment.

Cells were treated with 50 μg/ml cycloheximide for the indicated times followed by Western blot analysis or treated with 10 μM MG132 followed by denaturing IP. WT HEK293 cells treated with 100 nM thapsigargin for 4 hours were included as positive controls for UPR.

### Statistics.

Statistics tests were performed using GraphPad Prism, version 8.0 (GraphPad Software). Unless indicated otherwise, values are represented as means ± SEM. All experiments were repeated at least 2 to 3 times and/or performed with multiple independent biological samples from which representative data are shown. All data sets passed normality and equal variance tests. Statistical differences between the groups were compared using unpaired 2-tailed Student’s *t* test for 2 groups or 1-way ANOVA or 2-way ANOVA for multiple groups. *P* < 0.05 was considered statistically significant.

The intensities of the Western blot bands between different samples in some experiments were also statistically compared using 1-way ANOVA with post hoc Tukey-Kramer test in the R environment. The input data were first examined for homoscedasticity using the Breusch-Pagan test implemented in the ncvTest function in the R car package. In our experience, data that do not satisfy a constant variance usually display log-normal distribution. Therefore, the log-transformed data were used as input in those cases.

### Study approval.

Study protocols and written, informed consent protocols were approved by the institutional review boards at the Research Advisory Council (RAC) (King Faisal Specialist Hospital and Research Centre, KFSHRC RAC 2080006); the APHP-Délégation Interrégionale à la Recherche Clinique (DIRC) Assistance Publique-Hôpitaux de Paris, Paris, France (2015-03-03/DC 2014–2272); the Ethical Committee of the University of Naples Federico II (48/16), the University of Michigan Medical School (IRBMED, HUM00227482), and Health Sciences Research (IRB-HSR, University of Virginia, HSR230351). The patients and/or the parents provided written, informed consent prior to participation in the study. Written, informed consent was received for the use of the photographs.

### Data availability.

The materials and reagents used are either commercially available or are available upon request. All data and materials for the manuscript are described in Methods. Values for all data points in graphs are reported in the Supporting Data Values file.

## Author contributions

HHW and LLL designed and performed most experiments. ZJL generated all KI cells. XW performed structural analysis. OA, GC, MOH, LH, AM, MA, FSA, CB, KP, and NBP performed exome sequencing WES analysis, identified variants, and acquired clinical data. QP, SS, and MB provided insightful discussions. YL provided assistance on statistical analysis. SS and LQ directed the study, designed experiments, and wrote the manuscript with help from HHW, LLL, and ZJL. HHW, LLL, and ZJL wrote the Methods and figure legends. All authors commented on and approved the manuscript.

## Supplementary Material

Supplemental data

Supplemental video 1

Supplemental video 2

Supplemental video 3

Supporting data values

## Figures and Tables

**Table 1:**
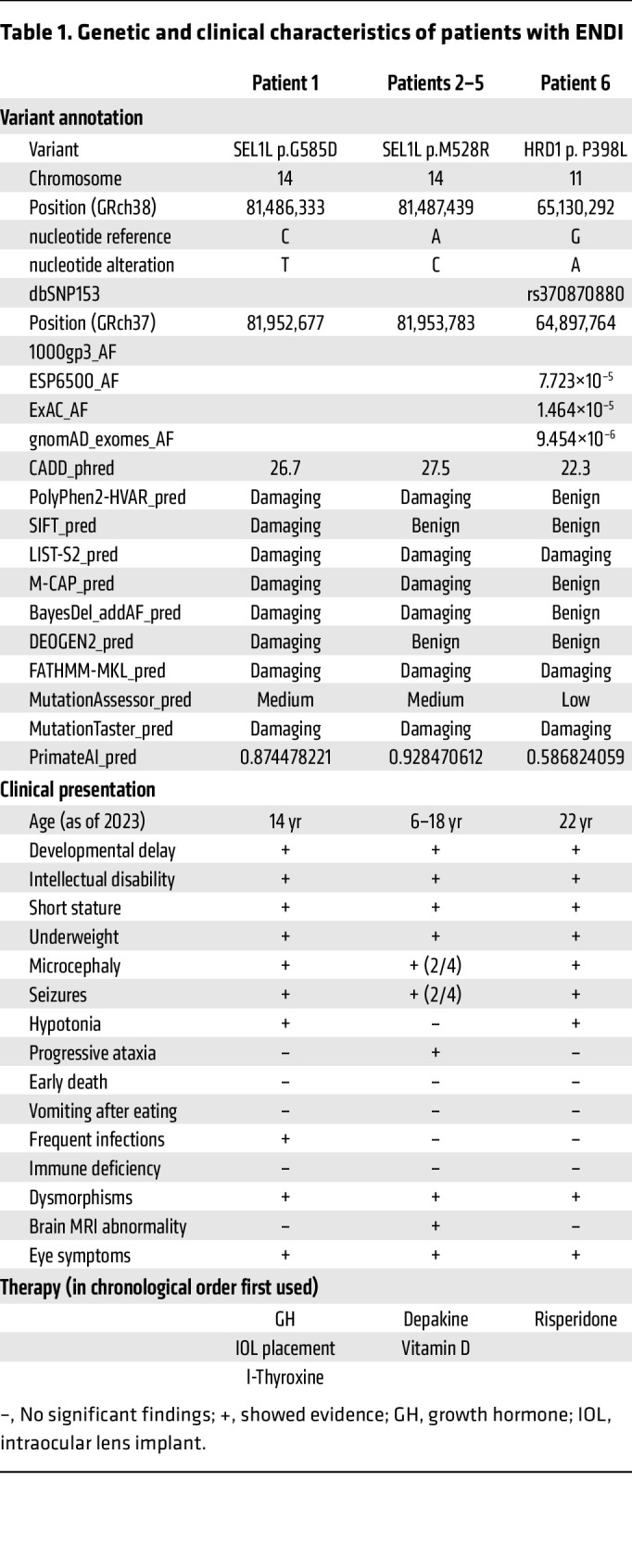
Genetic and clinical characteristics of patients with ENDI

## Version 1 11/09/2023

In-Press Preview

## Version 2 01/16/2024

Electronic publication
